# Supplementary material for: Study on the Aqueous CdTe Quantum Dots Solar Device Deposited by Blade Coating on Magnesium Zinc Oxide Window Layer
Source: Nanomaterials (Basel). 2022 Apr 30;12(9):1523. doi: 10.3390/nano12091523 (PMC9099490; doi:10.3390/nano12091523)
Supplement: Supplementary file 1 [file nanomaterials-12-01523-s001.zip › nanomaterials-1700742-supplementary.pdf]

nanomaterials

MDPI

Supplementary Materials

Study on the Aqueous CdTe Quantum Dots Solar Device Deposited by Blade Coating on Magnesium Zinc Oxide Window Layer

Bin Lv [1,2,3,*], Xia Liu [1,3], Bo Yan [1,3,*], Juan Deng [1,3], Fan Gao [1,3], Naibo Chen [1,3] and Xiaoshan Wu [2,4]

[1] Collaborative Innovation Center for Bio-Med Physics Information Technology of ZJUT, Zhejiang University of Technology, Hangzhou 310023, China; 2111909018@zjut.edu.cn (X.L.); jdeng@zjut.edu.cn (J.D.); gaofan@zjut.edu.cn (F.G.); chennb@zjut.edu.cn (N.C.)
[2] Nantong-Nanjing University Institute of Materials Engineering & Technology, Nantong 226019, China; xswu@nju.edu.cn
[3] Department of Applied Physics, Zhejiang University of Technology, Hangzhou 310023, China
[4] National Laboratory of Solid-State Microstructures, Nanjing University, Nanjing 210093, China
* Correspondence: binlv@zjut.edu.cn (B.L.); boyan@zjut.edu.cn (B.Y.)

Abstract: Aqueous CdTe quantum dots solar cells have been successfully fabricated by the blade coating method on the magnesium zinc oxide ($Zn_{1-x}Mg_xO$ or ZMO) window layer. Compared with the ZMO mono-window layer, the ZMO/CdS bi-window layer can decrease the interface recombination effectively due to the lower lattice mismatch and fast interdiffusion between CdS and CdTe. Moreover, the high temperature annealing of the CdTe quantum dots absorbed layer passivates the grain boundary of the CdTe crystalline via the replacement reaction of tellurium with sulfur. Finally, the conversion efficiency of our aqueous CdTe quantum dots solar device is improved from 3.21% to 8.06% with the introduction of the CdS interlayer and high temperature $CdCl_2$ annealing. Our aqueous CdTe quantum dots solar devices show a large open circuit voltage and fill factor which are comparable with the conventional devices that are fabricated with organic CdTe quantum dots. We believe that it is the spike-like conduction band alignment between the ZMO and CdTe absorbed layer that reduces the majority carrier concentration, leading to the decrease in interface recombination probability.

Keywords: aqueous CdTe quantum dots; solar cell; blade coating; $Zn_{1-x}Mg_xO$

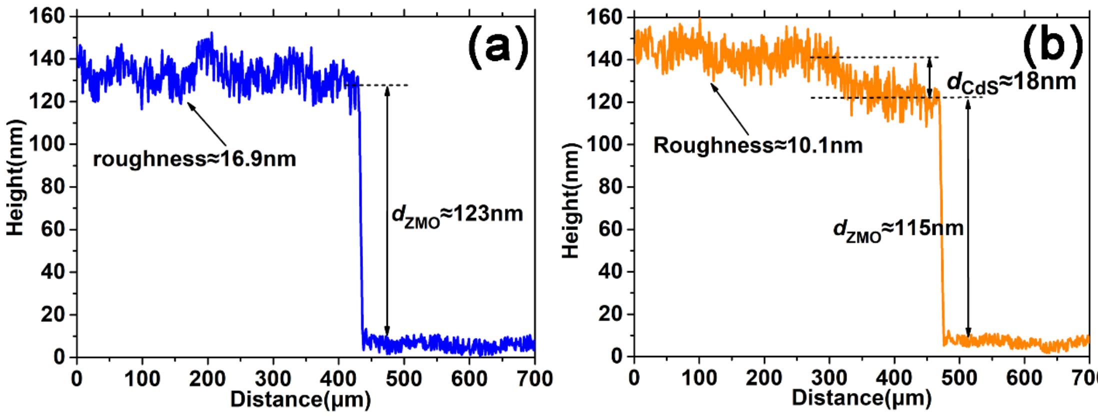

Figure S1. The thickness of (**a**) ZMO mono-window layer and (**b**) ZMO/CdS bi-window layer films investigated by stylus surface profilometry.

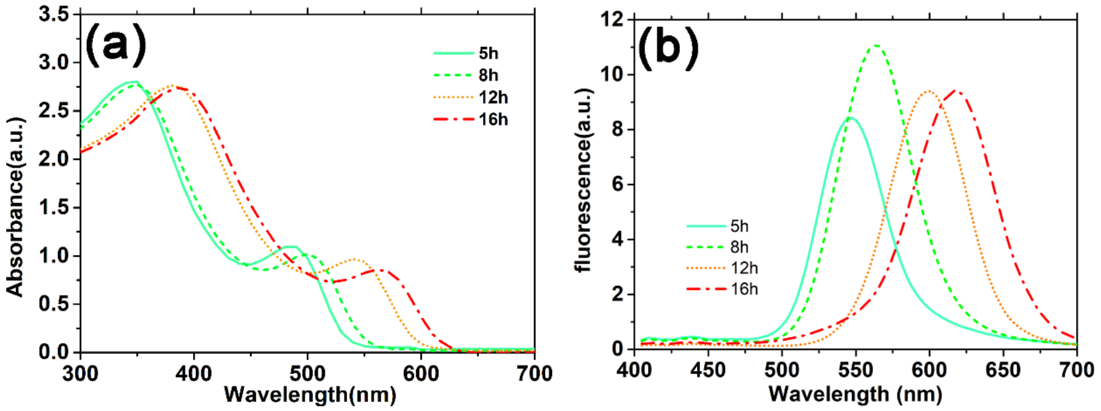

Figure S2. The (**a**) absorption and (**b**) photoluminescence spectra of CdTe QDs as functions of reflux time.

The evolution of both absorption and photoluminescence (PL) spectra of CdTe QDs as function of reflux time are presented in Figure S2a,b. With addition of NaHTe solution at room temperature, the color of the reaction mixture changes to lemon yellow instantly, indicating the beginning of nucleation. After about 2 h, CdTe QDs are formed with the color of the solution changing to golden yellow, and a weak cyan emission at 525 nm can be observed. As the reflux proceeds, the size of CdTe QDs keeps increasing and both the PL emission peaks and the absorption edges shift monotonously to longer wavelength. The PL peak position can approach 546, 562, 598 and 618 nm in the period of growth time of 5, 8, 12 and 16 h, respectively. All dispersion shows a sharp first excitonic absorption edge corresponding to the quantum confinement effect (QCE).

As is well known, the growth of QDs obeys the Ostwald ripening (OR) mechanism which is a thermodynamic process. The small QDs generated at first are thermodynamically unstable and decompose quickly to help the larger QDs reach a stable size. Thus, the size of QDs increases with increasing reflux time. Both the first excitonic absorption peak in the absorption spectra and emission peak in PL spectra show the red shift due to the weaken of QCE with increasing QDs size.
